# Protective effects of silibinin on LPS-induced inflammation in human periodontal ligament cells

**DOI:** 10.3389/fchem.2022.1019663

**Published:** 2022-10-10

**Authors:** Di Meng, Yuling Wang, Tongjun Liu

**Affiliations:** ^1^ Department of Stomatology, The Central Hospital Affilliated to Shandong First Medical University, Jinan, China; ^2^ Department of Stomatology, Shandong Qianfoshan Hospital, Jinan, China

**Keywords:** silibinin, inflammation, protective effects, LPS-induced, human periodontal ligament cells

## Abstract

Clinically, periodontitis is a chronic nonspecific inflammation that leads to damaged teeth and their supporting gum tissues. Although many studies on periodontitis have been conducted, therapy with natural products is still rare. Silibinin has been proven to have anti-inflammatory and antioxidant activities. However, the effects of silibinin on lipopolyssacharide (LPS)-induced inflammation in periodontal ligaments (PDLs) have not yet been investigated. In this study, the PDLs were treated with silibinin (10, 20, and 40 μM) in the presence of LPS. The results showed that silibinin treatment reduced the levels of NO, PGE_2_, IL-6, TNF-α, MMP-1, and MMP-3 and enhanced the activities of superoxide dismutase (SOD) and glutathione (GSH). Moreover, silibinin treatment downregulated RANKL levels and upregulated OPG and ALP levels. In summary, silibinin protected PDLs against LPS-induced inflammation, oxidative stress, and osteogenic differentiation.

## Introduction

Periodontitis is a chronic nonspecific inflammation caused by periodontal pathogenic bacteria (Seo et al., 2004; Nagatomo et al., 2006; Yamamoto et al., 2006). In the early stages of periodontitis, only the gums are inflamed, and bleed (Choi et al., 2012; Jun et al., 2012). However, with continuous stimulation of pathogenic microorganisms and their metabolites, the periodontal tissue produces immune responses, resulting in the secretion of a large number of inflammatory factors (Kim et al., 2009; Lee et al., 2012; Lei et al., 2014). These factors damage the periodontal supporting tissue, loosening the teeth, ultimately leading to tooth loss. The periodontal ligament (PDL) is an important periodontal tissue that connects the alveolar bone and root (Grzesik and Narayanan, 2002; Choi et al., 2012; Shin et al., 2015). PDL cells, the base units of PDLs, maintain periodontal health by secreting various inflammatory factors and osteoblast/osteoclast regulators (Abiko et al., 1998; Miura et al., 2000; Gyawali and Bhattarai, 2017).

Periodontitis is mainly caused by the imbalance between host’s defense and accumulating bacteria (Slots et al., 1986; Birkedal-Hansen, 1993). Lipopolysaccharides (LPS) are bacterial membrane proteins that are present in most subgingival Gram-negative organisms (Aznar et al., 1990; Nair et al., 1996). LPS is a stimulant that induces vascular dilatation and edema of periodontal tissues. In addition, sustained LPS stimulation damages periodontal tissue by producing harmful pro-inflammatory mediators, including IL-1β, IL-6, and TNF-α (Gowen et al., 1983; Boyce et al., 1989; Milica et al., 2017). Moreover, LPS stimulation increases the receptor activator of the nuclear factor kappa-B (NF-κB) ligand (RANKL) and reduces osteoprotegerin (OPG). These mediators further stimulate periodontitis (Belibasakis et al., 2007). Thence, clearing inflammation had been recognized as an effective method for improving disease.

**FIGURE 1:**
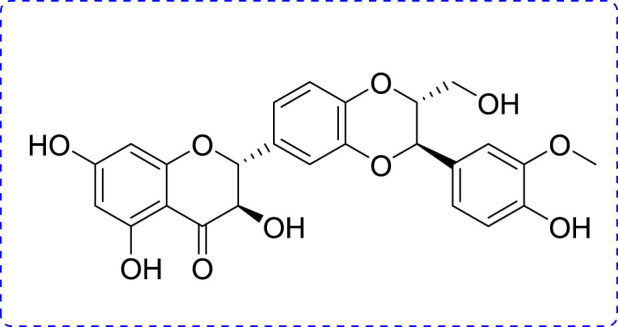
Chemical structure of SB.

Phytoconstituents have been used as beneficial and therapeutic agents since ancient times owing to their low toxicity and biological benefits. Some of them have beneficial therapeutic effects in the treatment of periodontitis. Silibinin (SB) is an important polyphenol found in *Silybum marianum L.* (Kim et al., 2003; Esmaeil et al., 2017; Amato et al., 2019) (Figure 1). Natural products and their derivatives play increasing roles in disease prevention (Cheng et al., 2022; Zhang et al., 2022). SB has been confirmed to have stimulating health benefits and shows promising biological activities, including anti-inflammatory, antioxidant, anti-tumor, and anti-fibrotic effects (Raina et al., 2013; Federico et al., 2017; Zheng et al., 2017). As a reliever of inflammation, SB reportedly ameliorates silica-induced pulmonary fibrosis by reducing the pro-inflammatory mediators (IL-1β, IL-6, and TNF-α) and collagen deposition (Ali et al., 2021). SB is effective against LPS-induced inflammation in PBMCs in horses (Gugliandolo et al., 2020). SB also ameliorates hepatotoxicity by inhibiting inflammation and oxidative stress (Saxena et al., 2022). Moreover, SB can enhance anti-inflammatory activity when combined with thymol (Chen et al., 2020), while it is also used as a beneficial dietary supplement to maintain body health and treat liver disorders.

The aforementioned evidence suggested that SB has good anti-inflammatory activity. Similarly, many studies have shown that periodontitis can be improved by inhibiting inflammatory responses. We designed and evaluated the anti-inflammatory effects of SB on LPS-induced hPDLCs.

## Results and discussion

### Cytotoxicity assay of SB

**FIGURE 2:**
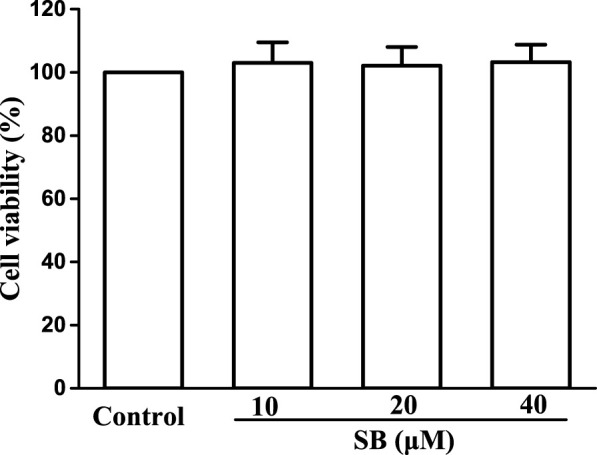
Cytotoxicity assay of SB.

To evaluate the cytotoxicity of SB on hPDLs, we exposed hPDLs to various concentrations of SB (10, 20, and 40 μM) for 24 h and tested cell viability using the MTT method. Based on the MTT assay results (Figure 2), SB was found to have no effect on the cell viability, indicating non-cytotoxicity to hPDLs at the tested concentrations (10–40 μM).

### SB reduce LPS-induced NO and PGE_2_

**FIGURE 3:**
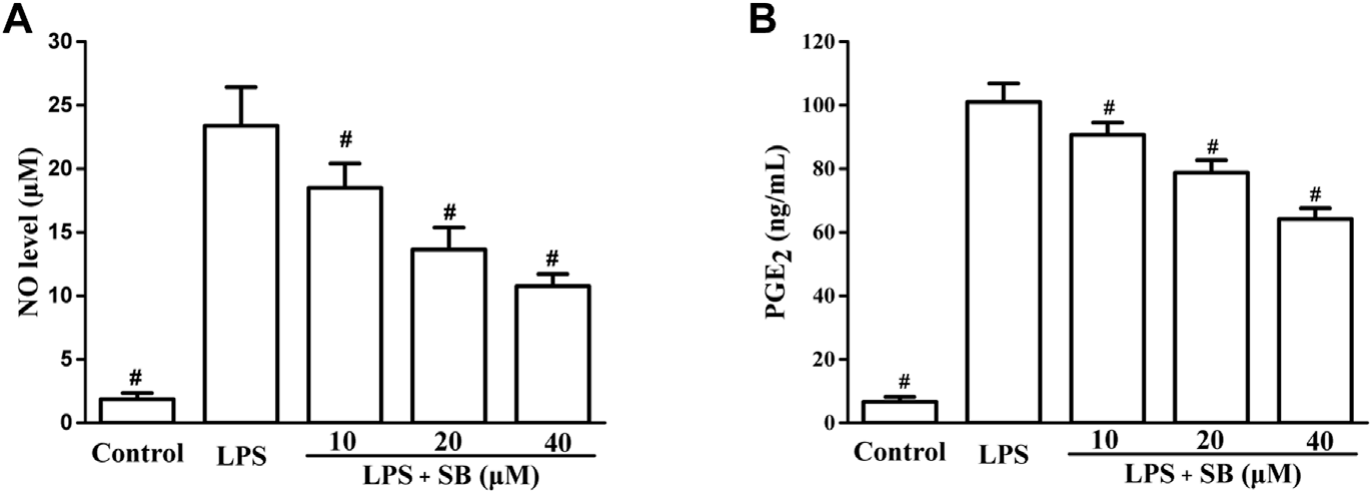
SB treatment reduced LPS-induced NO **(A)** and PGE_2_ **(B)** in hPDLs. ^#^ *p* < 0.05, compared to the LPS group.

NO and PGE_2_ are two inflammatory mediators produced by the induction of iNOS and COX-2, respectively (Jeong et al., 2009; Jeong et al., 2011). They can effectively influence inflammation and are classical markers of inflammation. Inhibition of NO and PGE_2_ is considered an effective strategy for the treatment of inflammation. The effects of SB on NO and PGE_2_ levels were assayed in LPS-induced hPDLs. From Figure 3A, it could be seen that LPS treatment significantly increased the NO level to 23.37 ± 3.04 μM compared to the control group. However, the elevated LPS-induced NO levels decreased by treatment with SB in a dose-dependent manner. The NO level reduced to 10.75 ± 0.96 μM, when treated with SB at 40 μM. Similarly, SB (40 μM) treatment inhibited the abnormally elevated PGE_2_ level induced by LPS stimulation to 64.12 ± 3.43 ng/ml (Figure 3B).

### SB inhibit LPS-induced IL-6 and TNF-α

**FIGURE 4:**
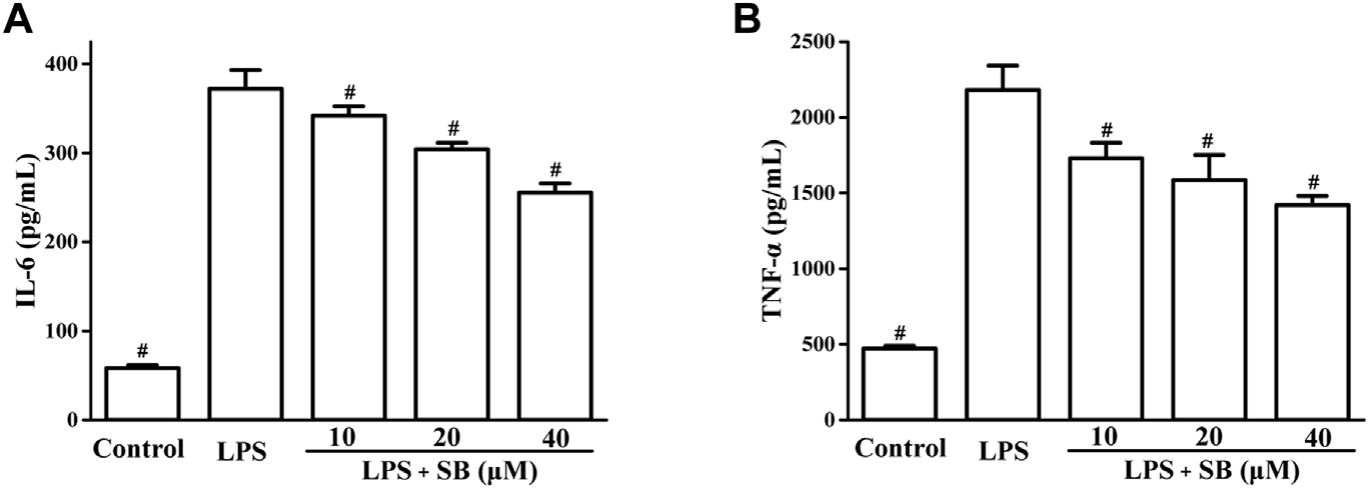
SB treatment inhibited LPS-induced IL-6 **(A)** and TNF-α **(B)** in hPDLs. ^#^ *p* < 0.05, compared to the LPS group.

Next, the effects of SB on LPS-induced IL-6 and TNF-α levels were examined by ELISA. It is well known that the overexpression of pro-inflammatory cytokines is closely related to various inflammatory processes (Lee et al., 2020; Tan et al., 2021). The release of pro-inflammatory cytokines results in the elimination of foreign pathogens. Therefore, reduction in pro-inflammatory cytokines is very important for the treatment of inflammation. As shown in Figure 4, LPS stimulation visibly increased IL-6 (up to 371.88 ± 21.13 pg/ml) and TNF-α (2,180.74 ± 160.30 pg/ml) levels compared to the control group. SB pre-treatment could significantly decrease the IL-6 level to 255.26 ± 10.39 pg/ml at 40 μM compared to the LPS-induced group (Figure 4A). Moreover, pre-treatment with 40 μM SB also reduced the TNF-α level to 1,419.61 ± 59.69 pg/ml (Figure 4B).

### SB inhibit LPS-induced MMP-1 and MMP-3

**FIGURE 5:**
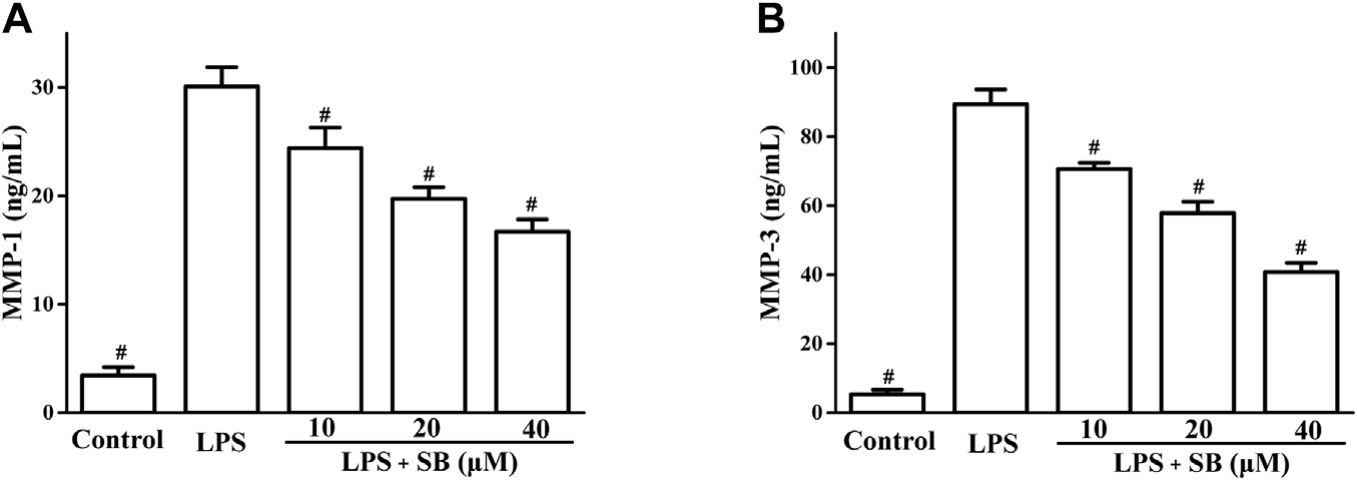
SB treatment inhibited LPS-induced MMP-1 **(A)** and MMP-3 **(B)** in hPDLs. ^#^ *p* < 0.05, compared to the LPS group.

Matrix metalloproteases (MMPs) are the major proteases of ECM metabolism and are involved in the destruction of periodontal tissues (Hosokawa et al., 2021). MMP-1 progresses and damages periodontal soft tissues by degrading type 1 collagen of periodontal tissues. MMP-3 is also reported to be involved in soft tissue destruction through the activation of pro-MMP-1. Hence, regulation of MMP-1 and MMP-3 leads to the improvement of periodontitis. SB treatment decreased LPS-induced MMP-1 and MMP-3 production in a dose-dependent manner (Figure 5). SB (40 μM) treatment reduced the MMP-1 and MMP-3 levels to 16.71 ± 1.12 and 40.72 ± 2.72 ng/ml, respectively, compared to the LPS group (30.09 ± 1.76 and 89.41 ± 4.23 ng/mL pg/ml, respectively).

### SB regulate LPS-induced SOD and GSH

**FIGURE 6:**
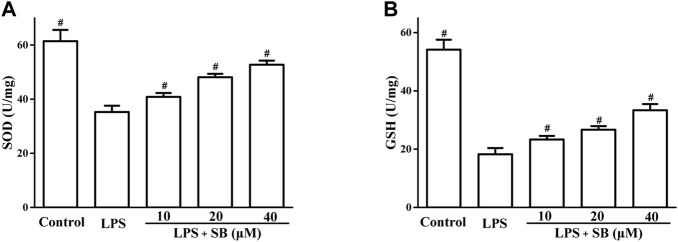
SB treatment regulated LPS-induced SOD **(A)** and GSH **(B)** in hPDLs. ^#^ *p* < 0.05, compared to the LPS group.

It has been revealed that the inflammatory response involves cross-talk with oxidative stress in the defense against pathogenic microorganisms (Chang et al., 2014; Wang et al., 2019). The effects of SB on superoxide dismutase (SOD) and glutathione (GSH) levels, which are important indicators of oxidative stress, were assayed. The results in Figure 6 showed that LPS stimulation could obviously reduce SB on SOD levels in hPDLs, which could be increased by SB treatment (Figure 5A). Similarly, treatment with SB (Figure 6A) significantly increased GSH reduction following LPS stimulation (Figure 6B).

### SB regulate LPS-induced RANKL and OPG

**FIGURE 7:**
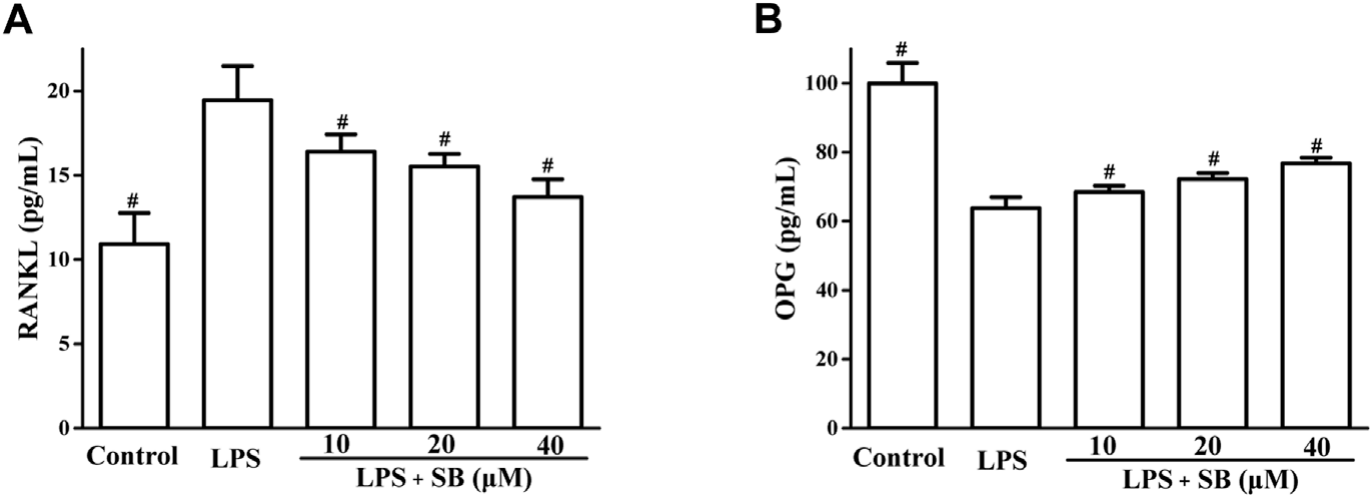
SB treatment regulated LPS-induced SOD **(A)** and GSH **(B)** in hPDLs. ^#^ *p* < 0.05, compared to the LPS group.

RANKL and OPG have been reported to play important roles in bone resorption. RANKL regulates osteoclast differentiation (Shu et al., 2008). OPG is a decoy receptor that binds to RANKL to regulate its activity (Bae et al., 2018). We evaluated the effects of SB on LPS-induced RANKL and OPG expressions. As shown in Figure 7A, SB treatment clearly downregulated the unusually high RANKL expression induced by LPS. However, treatment with SB enhanced the unusually low OPG levels induced by LPS (Figure 7B).

### SB regulate LPS-induced ALP

**FIGURE 8:**
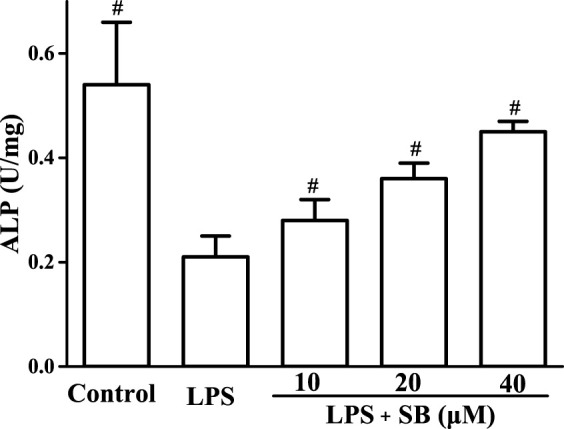
SB treatment regulated LPS-induced ALP in hPDLs. ^#^ *p* < 0.05, compared to the LPS group.

Alkaline phosphatase (ALP) is an important marker of osteoblast differentiation and plays a key role in connective tissue calcification and mineral deposits (Li and Peng, 2019). Studies have shown that LPS can inhibit ALP activity, cell metabolism, and viability in osteoblasts. Our results (Figure 8) showed that LPS treatment significantly inhibited ALP activity compared with the control group. However, the reduced ALP activity induced by LPS treatment was effectively reversed by treatment with SB.

## Conclusion

**FIGURE 9:**
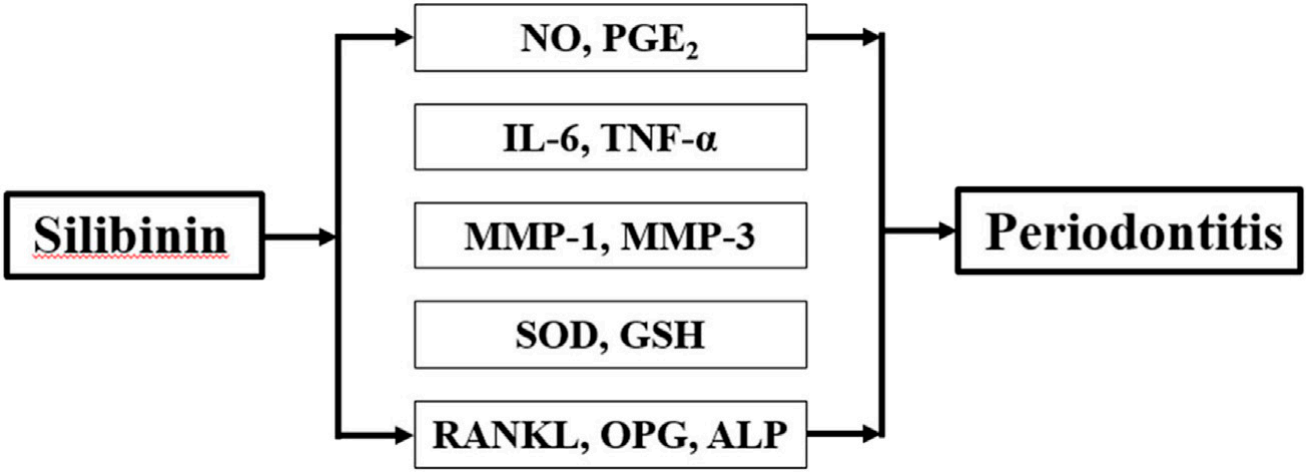
Effect of SB on LPS-induced hPDLs.

We treated PDLs with silibinin (10, 20, and 40 μM) in the presence of LPS to investigate the protective effects of silibinin against periodontitis. Our findings revealed that silibinin treatment reduced the levels of NO, PGE_2_, IL-6, TNF-α, MMP-1, and MMP-3 and enhanced the activities of SOD and GSH. Moreover, silibinin treatment downregulated RANKL levels and upregulated OPG and ALP levels. Our results indicate that silibinin could affect inflammation, oxidative stress, and osteogenic differentiation capacity against LPS (Figure 9) and could be used as an effective agent for the treatment of periodontitis.

## Experimental

### Cell culture and treatment

hPDLCs were prepared using previously reported methods (Blufstein et al., 2021) and cultured in α-MEM with 10% FBS, 100 U/mL penicillin, and 100 μg/ml. The cells were divided into five groups: control group (no agent), LPS group (treatment with 1 μg/ml LPS), and three SB groups (treatment with 10, 20, and 40 μM SB, before 1 μg/ml LPS treatment).

### Cytotoxicity assay

The cytotoxicity of SB on hPDLCs was assayed using the MTT assay. hPDLCs were seeded into 96-well plates for 24 h and then treated with SB (10, 20, and 40 μM) for another 24 h. The MTT reagent (0.5 mg/ml) was added to each well and incubated for 4 h. DMSO was used to dissolve the resulting crystals, followed by absorbance measurement at 570 nm.

### Determination of NO

The hPDLCs were treated with SB (10, 20, and 40 μM) for 2 h, followed by exposure to LPS (1 μg/ml) for 24 h. The NO level in the supernatant was then determined using the Griess reagent. An equal volume of the Griess reagent was added to the culture supernatant and incubated for 10 min. The absorbance was then measured at 540 nm.

### Determination of PGE_2_

After hPDLCs were treated for 24 h, the culture supernatant was harvested. PGE2 levels in each group were measured using an EIA kit according to the manufacturer’s instructions.

### Determination of IL-6, TNF-α, MMP-1, MMP-3, and OPG

After hPDLCs were treated for 24 h, IL-6 and TNF-α levels were measured in the harvested culture supernatant using the corresponding IL-6, TNF-α, MMP-1, MMP-3, or OPG ELISA assay kits.

### Determination of SOD and GSH

After hPDLCs were treated for 24 h, SOD and GSH levels were measured in the harvested cells using the corresponding commercial kits.

### Determination of RANKL

After hPDLCs were treated for 24 h, the cells were harvested and lysed and RANKL levels were measured using RANKL ELISA kits.

### Determination of ALP activity

After hPDLCs were treated for 7 days, the harvested cells were lysed using 1% Triton X-100. After centrifugation, ALP activity of the supernatant was detected the ALP activity using an ALP assay kit.

## Data Availability

The datasets presented in this study can be found in online repositories. The names of the repository/repositories and accession number(s) can be found in the article/Supplementary Material.
